# Supplementary material for: Evolution of sequence traits of prion-like proteins linked to amyotrophic lateral sclerosis (ALS)
Source: PeerJ. 2022 Nov 17;10:e14417. doi: 10.7717/peerj.14417 (PMC9676014; doi:10.7717/peerj.14417)
Supplement: Supplemental Information 1 [file peerj-10-14417-s001.pdf]

Figure Legend:

Three phylogenetic trees of TDP43, EWSR1/TAF15/FUS and hnRNPA2 respectively.

The tree are labelled with the following attributes (from inside to outside):

(i) The aLRT support values from the PhyML program indicated with a coloured dot at the end of each branch. The colour key is on the right of the figure.

(ii) The identifiers of proteins (from the OrthoDB database), coloured according to clade: **orange** (animals), **green** (plants), **blue** (fungi) and white for others.

(iii) A barchart in **dark green** indicating number of RRM RNA-binding domains, except that for TDP43, it indicates the total number of domains (N-terminal domain + RRM domains). The x-axis of the bar-chart is shown at the break in the circular tree.

(iv) A barchart in **red** indicating the protein sequence length. The x-axis of the bar-chart is shown at the break in the circular tree.

(v) A barchart in **gold** indicating the proportion of intrinsic disorder in each sequence. The x-axis of the bar-chart is shown at the break in the circular tree.

(vi) A barchart in **dark blue** indicating the PLAAC prion-like PRD score in each sequence. The x-axis of the bar-chart is shown at the break in the circular tree.

(vii) A dot indicating whether the protein is predicted to be recruited to stress granules by the SGNN server, **red** for yes, **green** for no.

(viii) A diagram indicating proportionally where the RRM and NTD (N-terminal) RNA-binding domains are in the sequences (the N-terminus is on the inside). The colour key for the domains is at the side of the figure.

(ix) A diagram indicating proportionally where the PLAAC-annotated PRD prion-like domains are in the sequences (the N-terminus is on the inside). A colour code for the PRD prion-like score is on the right.

(x) A diagram indicating proportionally where the fLPS-annotated compositionally biased (CB) regions are in the sequences (the N-terminus is on the inside), using default fLPS parameters. Only the ten most common overall are listed. A colour code for the CB regions is on the right. The CB regions are named with the CB signature which is a list of the biasing residues in decreasing order of precedence in curly brackets.

(xi) A diagram indicating proportionally where the fLPS-annotated compositionally biased (CB) regions are in the sequences (the N-terminus is on the inside), **using parameters for annotating low-complexity regions *(–m 5 –M 25 –t 1e-06*).** Only the ten most common overall are listed. A colour code for the CB regions is on the right. The CB regions are named with the CB signature which is a list of the biasing residues in decreasing order of precedence in curly brackets.

Further annotations

Filled black circles denote the approximate extent of plants in the TDP43 and hnRNPA2 trees.
The black stars denote the approximate extent of the vertebrate clade in the TDP43 tree.

The largest clades containing >90% orthologs of FUS, TAF15 or EWSR1 are indicated with shading on the FUS/TAF15/EWSR1 tree.

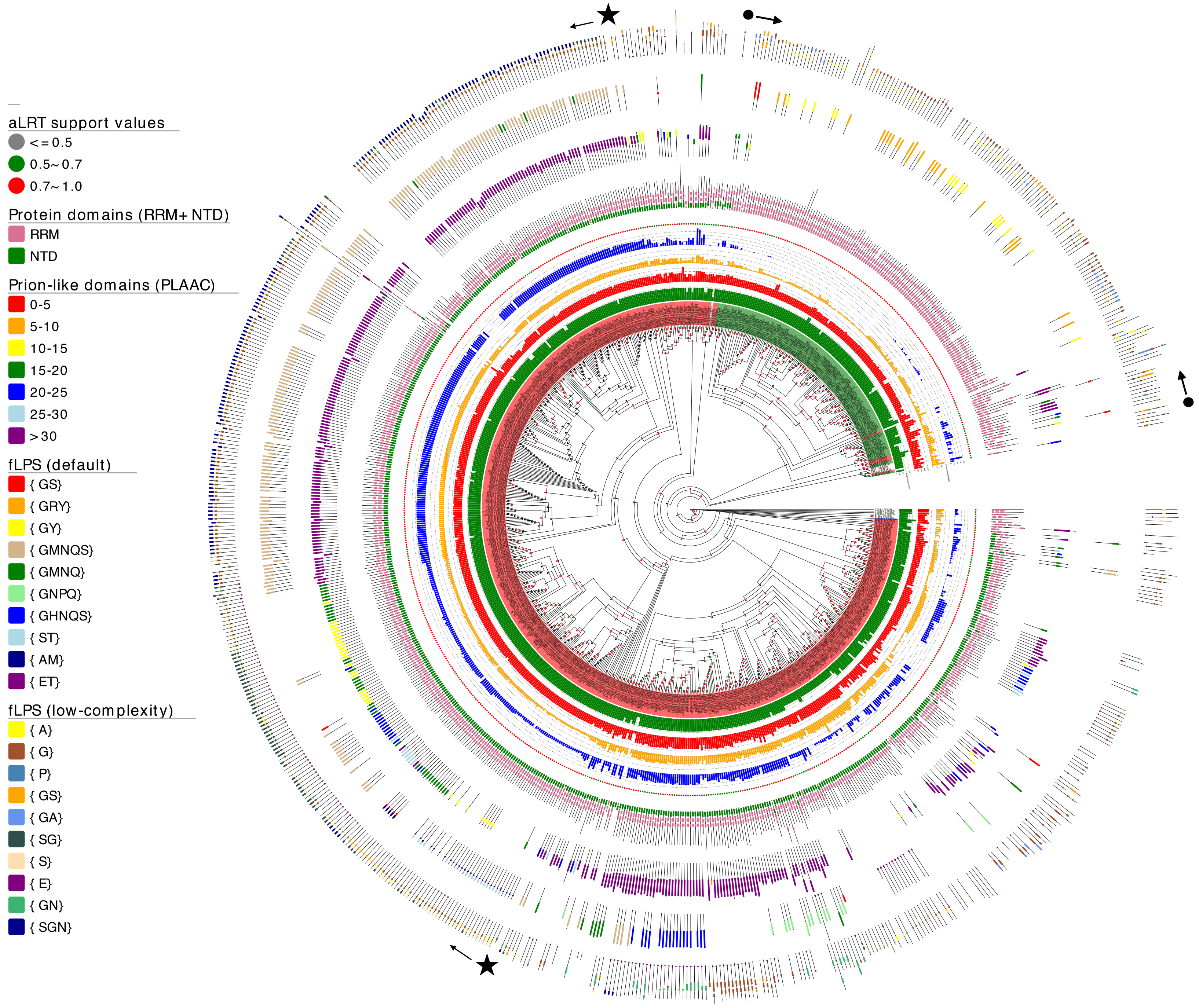
aLRT support values
<=0.5
0.5~0.7
0.7~1.0
Protein domains (RRM+NTD)
RRM
NTD
Prion-like domains (PLAAC)
0-5
5-10
10-15
15-20
20-25
25-30
>30
fLPS (default)
{GS}
{GRY}
{GY}
{GMNQS}
{GMNQ}
{GNPQ}
{GHNQS}
{ST}
{AM}
{ET}
fLPS (low-complexity)
{A}
{G}
{P}
{GS}
{GA}
{SG}
{S}
{E}
{GN}
{SGN}

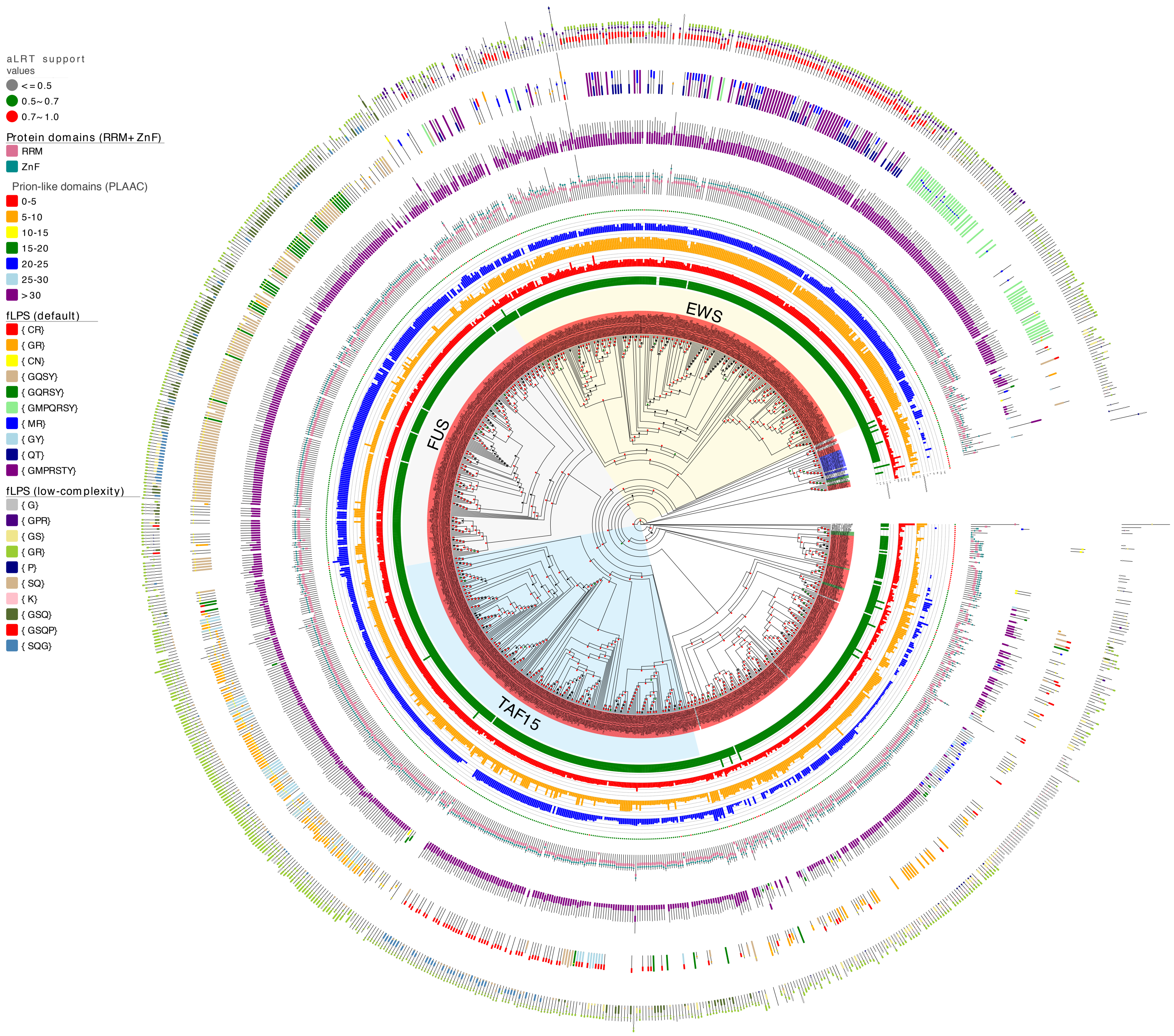
aLRT support values
<=0.5
0.5~0.7
0.7~1.0
Protein domains (RRM+ ZnF)
RRM
ZnF
Prion-like domains (PLAAC)
0-5
5-10
10-15
15-20
20-25
25-30
>30
fLPS (default)
{CR}
{GR}
{CN}
{GQSY}
{GQRSY}
{GMPQRSY}
{MR}
{GY}
{QT}
{GMPRSTY}
fLPS (low-complexity)
{G}
{GPR}
{GS}
{GR}
{P}
{SQ}
{K}
{GSQ}
{GSQP}
{SQG}
EWS
FUS
TAF15

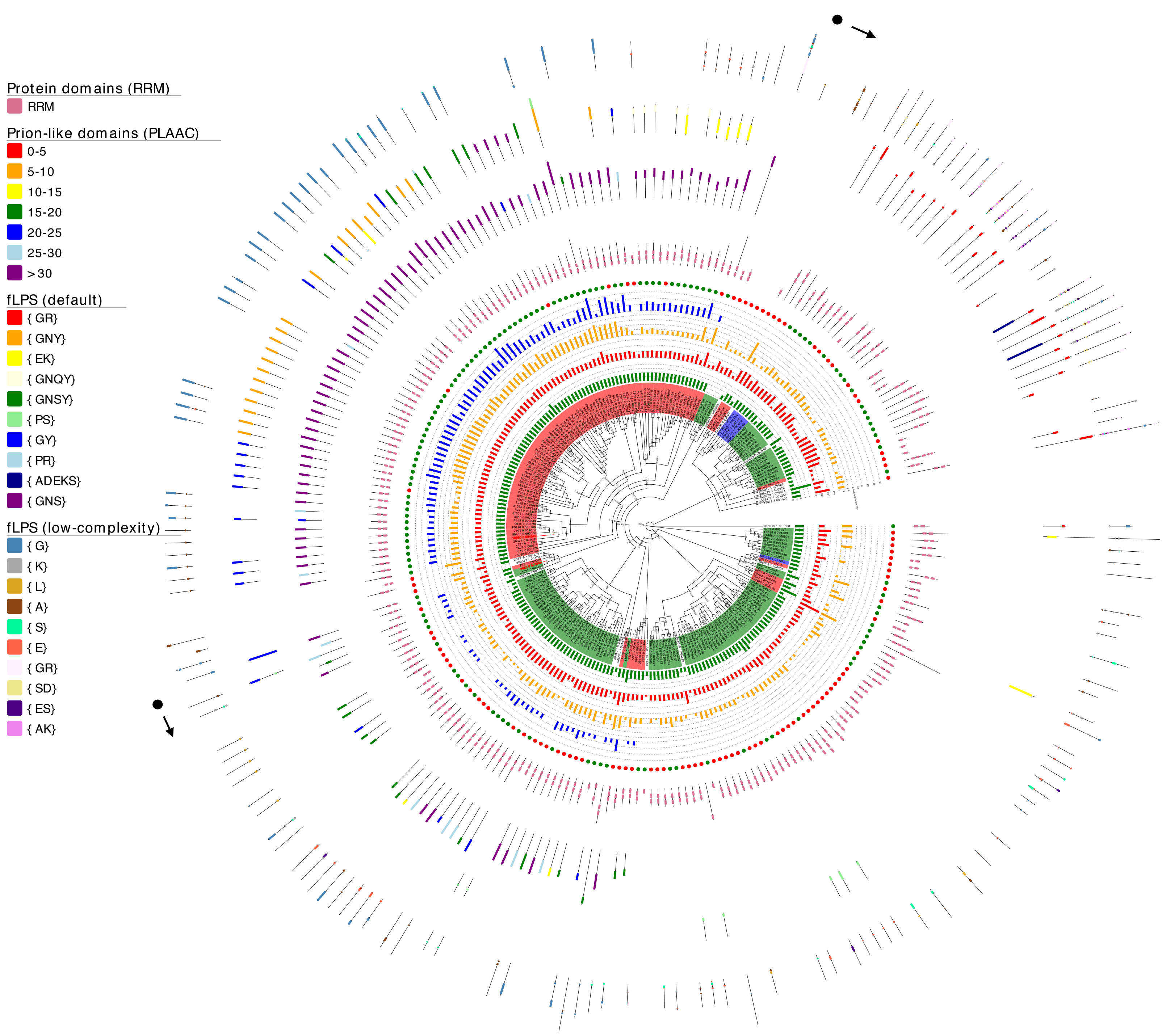
Protein domains (RRM)
RRM
Prion-like domains (PLAAC)
0-5
5-10
10-15
15-20
20-25
25-30
>30
fLPS (default)
{ GR}
{ GNY}
{ EK}
{ GNQY}
{ GNSY}
{ PS}
{ GY}
{ PR}
{ ADEKS}
{ GNS}
fLPS (low-complexity)
{ G}
{ K}
{ L}
{ A}
{ S}
{ E}
{ GR}
{ SD}
{ ES}
{ AK}
