# Supplementary material for: Evolution of sequence traits of prion-like proteins linked to amyotrophic lateral sclerosis (ALS)
Source: PeerJ. 2022 Nov 17;10:e14417. doi: 10.7717/peerj.14417 (PMC9676014; doi:10.7717/peerj.14417)
Supplement: Supplemental Information 3 [file peerj-10-14417-s003.pdf]

Figure Legend:
Total counts of sequences containing single-residue CB regions with P $\leq 1 \times 10^{-4}$ for (A) FUS, (B) TAF15 and (C) EWSR1 orthologs. The CB regions are labelled {*x*}, where *x* is the biasing residue. These are counts of sequences, not of CB regions.

(A)

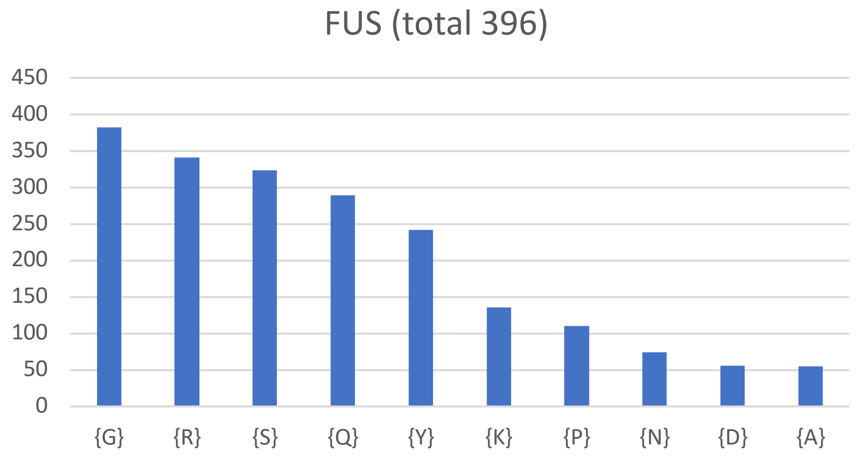
FUS (total 396)
450
400
350
300
250
200
150
100
50
0
{G}
{R}
{S}
{Q}
{Y}
{K}
{P}
{N}
{D}
{A}

(B)

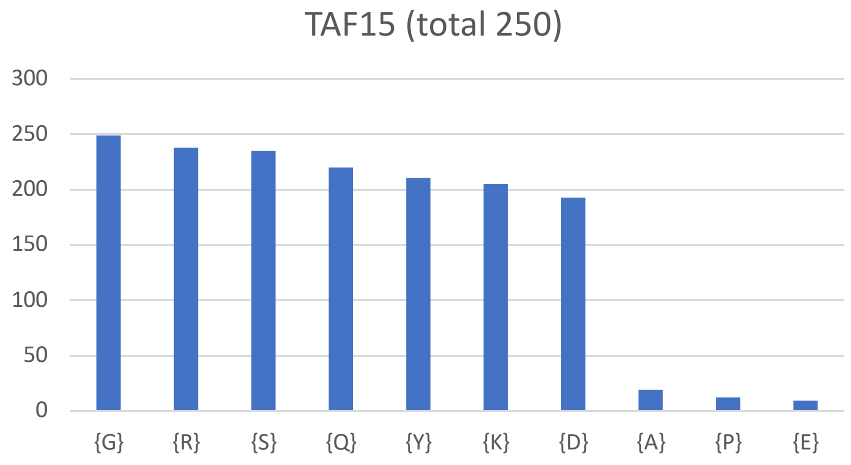
TAF15 (total 250)
300
250
200
150
100
50
0
{G}
{R}
{S}
{Q}
{Y}
{K}
{D}
{A}
{P}
{E}

(C)

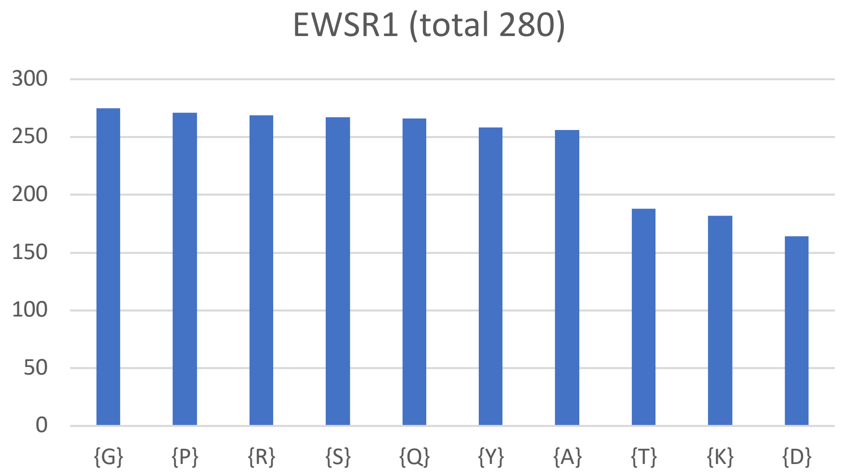
EWSR1 (total 280)
300
250
200
150
100
50
0
{G}
{P}
{R}
{S}
{Q}
{Y}
{A}
{T}
{K}
{D}
