# Supplementary material for: Evolution of sequence traits of prion-like proteins linked to amyotrophic lateral sclerosis (ALS)
Source: PeerJ. 2022 Nov 17;10:e14417. doi: 10.7717/peerj.14417 (PMC9676014; doi:10.7717/peerj.14417)
Supplement: Supplemental Information 4 [file peerj-10-14417-s004.pdf]

Figure Legend:

Plots of CB region length (in residues) versus –log(P-value) for the six most common single-residue biases for each of FUS, TAF15 and EWSR1 respectively. The plots for each protein are arrayed on one page each. The P-values are from the fLPS program.

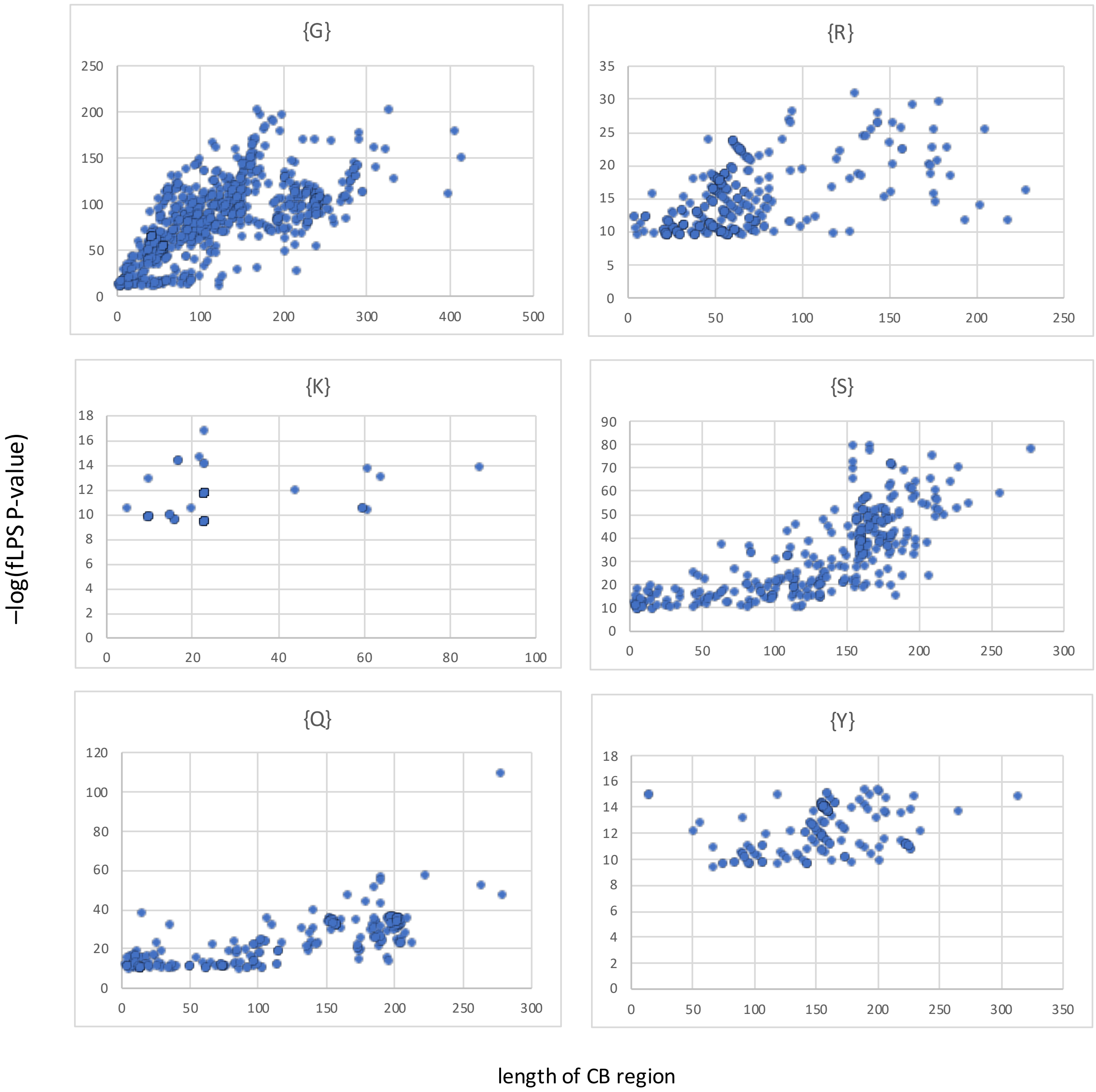

FUS

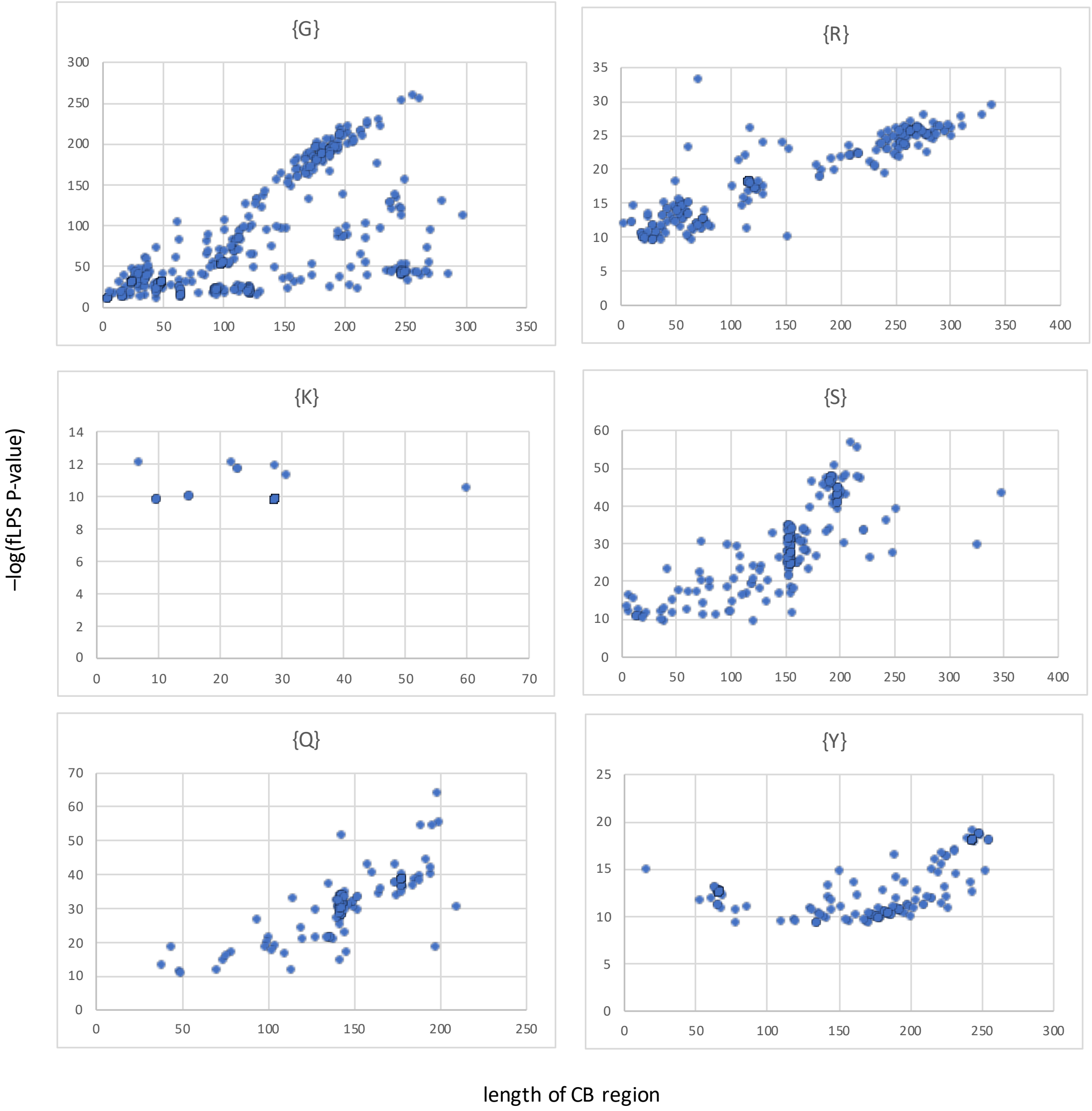

TAF15

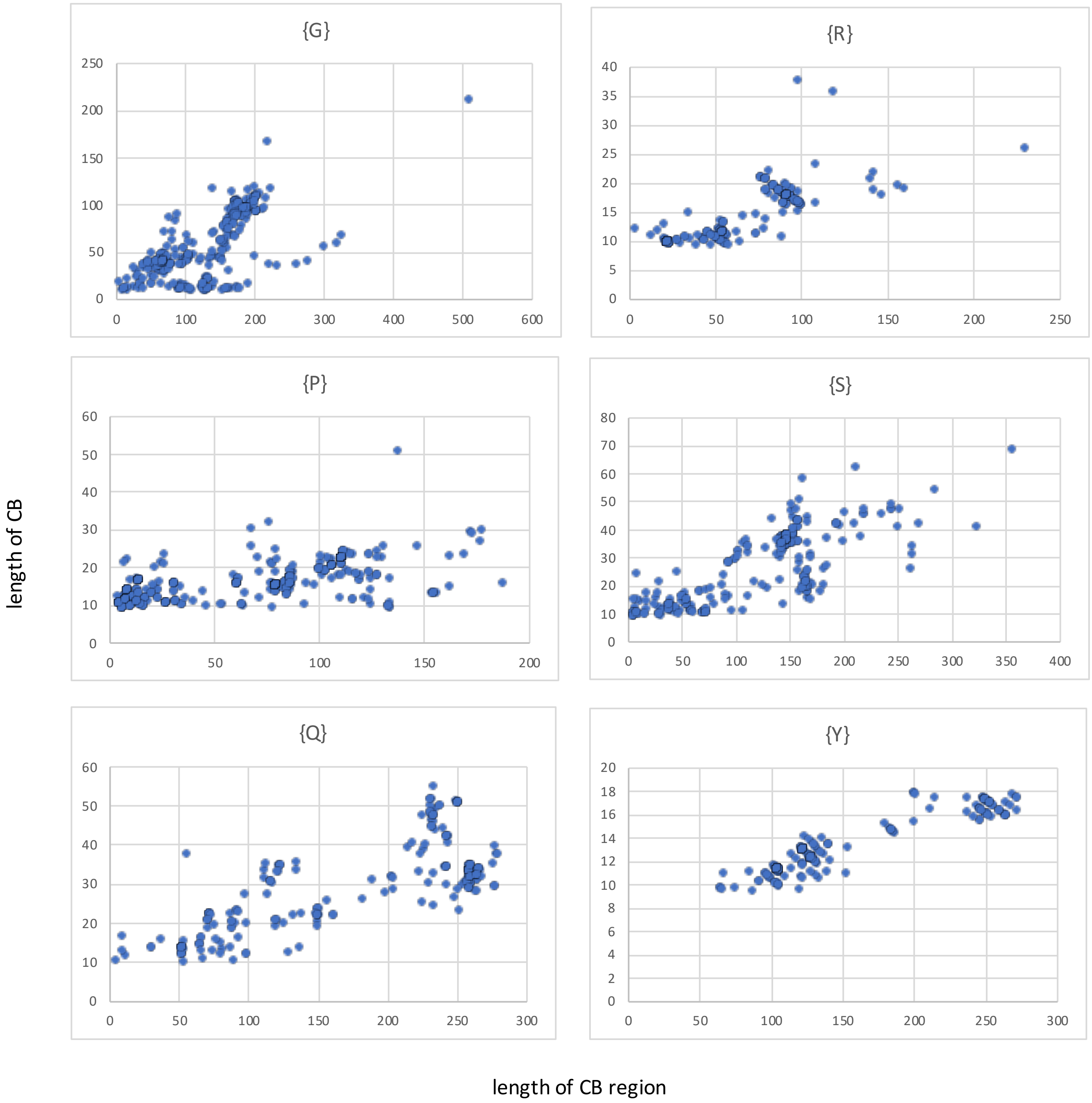

EWSR1
