# Supplementary material for: Evolution of sequence traits of prion-like proteins linked to amyotrophic lateral sclerosis (ALS)
Source: PeerJ. 2022 Nov 17;10:e14417. doi: 10.7717/peerj.14417 (PMC9676014; doi:10.7717/peerj.14417)
Supplement: Supplemental Information 5 [file peerj-10-14417-s005.pdf]

Figure Legend (figure overleaf): The top-ten most common low-complexity regions (LCRs) with fLPS P-value ≤1x10^{-6}, for the three trees, split into kingdoms. Within a protein family, LCRs that are not conserved across kingdoms are represented by grey LCRs. {G}-rich LCRs are labelled red, {GR}-rich gold, {P}-rich green and {A}-rich, with other types conserved across kingdoms coloured purple.

The total counts are on the y-axis and the bias signatures from the fLPS algorithm are on the x-axis. The panels are for the following data:

(A) The metazoan sequences from the EWSR1/TAF15/FUS tree (EWSR1, etc.);

(B) The non-metazoan sequences from the EWSR1/TAF15/FUS tree (EWSR1, etc.);

(C) The metazoan sequences from the TDP43 tree;

(D) The plant sequences from the TDP43 tree;

(E) The metazoan sequences from the hnRNPA2 tree;

(F) The fungi sequences from the hnRNPA2 tree;

(G) The plant sequences from the hnRNPA2 tree;

(H) The other (not fungi, metazoan or plant) sequences from the hnRNPA2 tree.

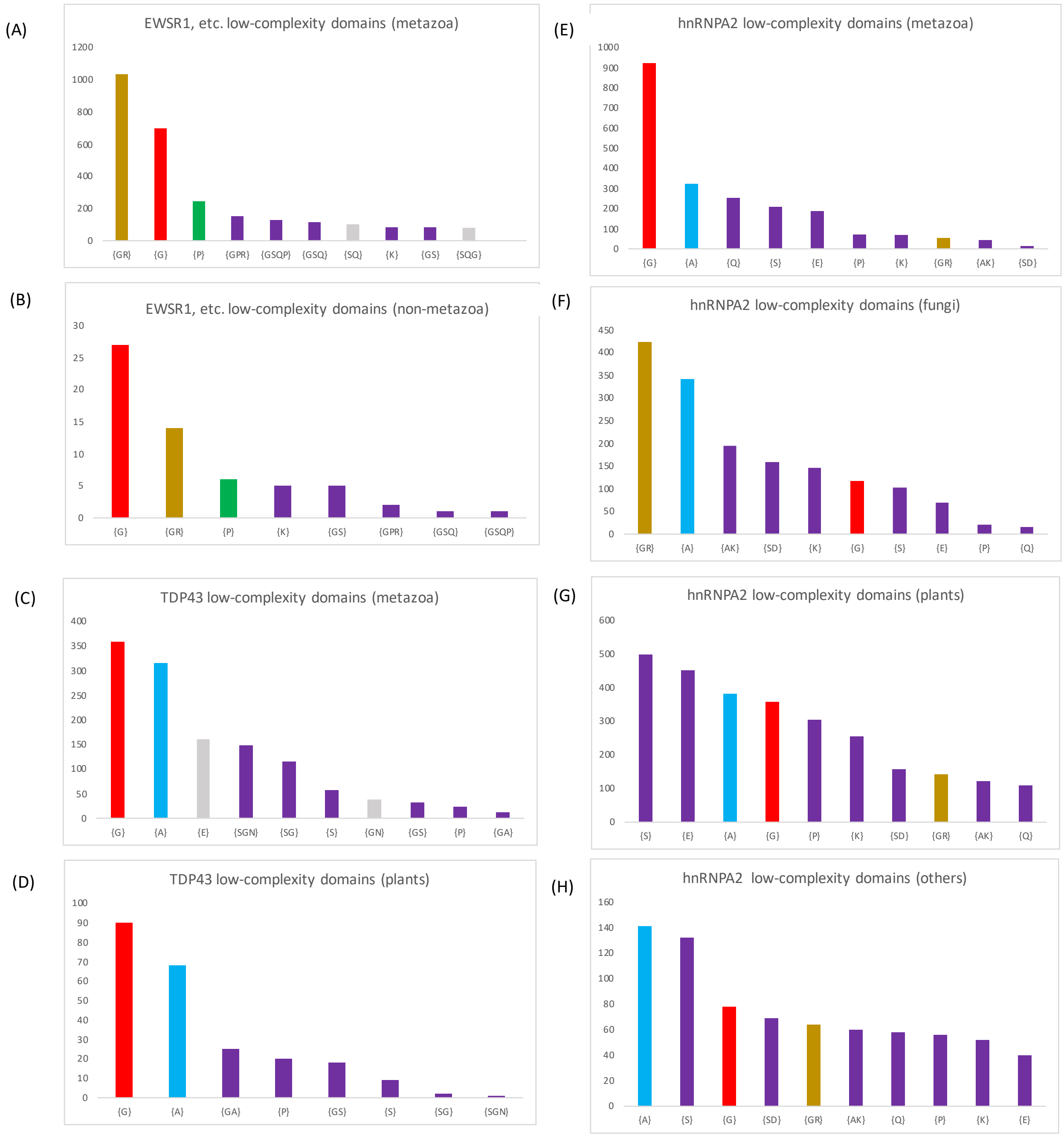
(A)
EWSR1, etc. low-complexity domains (metazoa)
0 200 400 600 800 1000 1200
{GR} {G} {P} {GPR} {GSQP} {GSQ} {SQ} {K} {GS} {SQG}
(B)
EWSR1, etc. low-complexity domains (non-metazoa)
0 5 10 15 20 25 30
{G} {GR} {P} {K} {GS} {GPR} {GSQ} {GSQP}
(C)
TDP43 low-complexity domains (metazoa)
0 50 100 150 200 250 300 350 400
{G} {A} {E} {SGN} {SG} {S} {GN} {GS} {P} {GA}
(D)
TDP43 low-complexity domains (plants)
0 10 20 30 40 50 60 70 80 90 100
{G} {A} {GA} {P} {GS} {S} {SG} {SGN}
(E)
hnRNPA2 low-complexity domains (metazoa)
0 100 200 300 400 500 600 700 800 900 1000
{G} {A} {Q} {S} {E} {P} {K} {GR} {AK} {SD}
(F)
hnRNPA2 low-complexity domains (fungi)
0 50 100 150 200 250 300 350 400 450
{GR} {A} {AK} {SD} {K} {G} {S} {E} {P} {Q}
(G)
hnRNPA2 low-complexity domains (plants)
0 100 200 300 400 500 600
{S} {E} {A} {G} {P} {K} {SD} {GR} {AK} {Q}
(H)
hnRNPA2 low-complexity domains (others)
0 20 40 60 80 100 120 140 160
{A} {S} {G} {SD} {GR} {AK} {Q} {P} {K} {E}
